# Buprenorphine for Children and Adolescents with Sickle Cell Disease: A Scoping Review

**DOI:** 10.3390/children13030388

**Published:** 2026-03-10

**Authors:** Joseph deBettencourt, Matthew Nagy, Chloe Rotman, Christine Greco, Charles Berde, Natasha M. Archer

**Affiliations:** 1 Dana-Farber/Boston Children’s Cancer and Blood Disorders Center, Harvard Medical School, Boston, MA 02115, USA; 2 Department of Pediatrics, Boston Children’s Hospital, Harvard Medical School, Boston, MA 02115, USA; 3 Medical Library, Boston Children’s Hospital, Boston, MA 02115, USA; 4 Department of Anesthesiology, Critical Care, and Pain Medicine, Boston Children’s Hospital, Harvard Medical School, Boston, MA 02115, USA

**Keywords:** sickle cell disease, pediatrics, buprenorphine, pain management, opioids

## Abstract

**Highlights:**

**What are the main findings?**

- There is limited pediatric specific data on the use of buprenorphine for managing sickle cell pain.
- Despite this limitation, there is growing interest in this medication, and small studies have shown it is effective for pain control and may potentially have an improved side-effect profile over full agonist μ-opioids.

**What are the implications of the main findings?**

- Further studies are required to understand the use and utility of buprenorphine for pediatric patients with sickle cell disease.
- Creation of guidelines for use of buprenorphine for children with sickle cell disease may expand use.

**Abstract:**

**Background and Objective:** Sickle cell disease (SCD) is an inherited blood disorder associated with recurrent painful crises. Sickle cell pain crises are a significant source of distress for patients and contribute substantially to hospital utilization among SCD populations. Many children with SCD also experience chronic pain, which is often multifactorial in nature. The management of both acute and chronic pain in SCD commonly relies on opioid medications. Acute and chronic use of opioids is associated with health risks and potential complications, which has raised interest in alternatives. Buprenorphine is a partial μ-receptor agonist with strong affinity that confers pain relief and may have an improved side-effect profile. While there is emerging evidence for its use in adult patients, the data is less developed in pediatrics. **Methods:** A scoping review was designed in accordance with PRISMA guidelines to systematically explore the literature on buprenorphine use in pain management for children with sickle cell disease (SCD). **Results:** This review shows that the published literature in this area is of low quality and extremely limited, and there is a lack of trials specifically designed to address the use of buprenorphine for this patient population. Studies are limited in their generalizability but suggest that buprenorphine may be useful in managing pain in this population. **Conclusions:** While promising, more data is required both retrospectively and prospectively to understand the utility of buprenorphine. The development of pediatric-specific protocols for transitioning from full µ-receptor agonist opioids to buprenorphine is also needed.

## 1. Introduction

Sickle cell disease (SCD) is an inherited blood disorder associated with recurrent painful crises [1]. Sickle cell pain crises are a significant source of distress for patients and families and contribute substantially to hospital utilization among SCD populations [2]. The primary management of these painful episodes traditionally involves opioid analgesics [3]. However, chronic opioid use is associated with complications such as tolerance, dependence, overdose risk, and negative impacts on quality of life, alongside increasing concerns about opioid stigma and barriers in care [3,4]. Buprenorphine, a partial μ-opioid receptor agonist and kappa antagonist, has emerged as a promising alternative with a safer side-effect profile, lesser respiratory depression risk, and potential for pain control in SCD [5,6]. Through its unique partial µ-opioid receptor agonism, buprenorphine confers pain relief without sustained receptor activation, which is thought to attenuate respiratory depression and reduce the development of opioid-induced sensitization relative to full agonist opioids [7,8]. This unique pharmacology also reduces symptoms associated with withdrawal, and it is thought that the risk of overdose from buprenorphine alone is low [9].

With this unique pharmacology, buprenorphine is now being used to treat pain in adult patients with SCD, and initial studies suggest efficacy and patient satisfaction with its use. An adult study of 36 patients who were transitioned to buprenorphine reported a 72.5% reduction in acute care utilization, with very few patients discontinuing treatment [5]. A real-world follow-up of this study with 32 patients continued to show decreased healthcare utilization, with most patients opting to continue treatment [10]. Importantly, patient themselves also report improvement in quality of life measures on buprenorphine, suggesting that beyond pain control, this medication may be beneficial for patients with sickle cell [11]. While buprenorphine use in adult SCD pain management is growing, evidence regarding its safety, effectiveness, and protocols for use in pediatric populations is significantly limited [12]. Pain management in pediatric patients is distinct from adult pain management both psychologically and physiologically, making it difficult to extrapolate adult data to pediatric patients [13]. Pediatric SCD patients, who are at increased risk of pulmonary complications compared to adults, may also derive increased benefit from the decreased risk of respiratory depression from buprenorphine. Examining the currently literature available is key to understanding use and future research on this topic [14].

Due to the limited data currently available on this topic and a lack of controlled trials, a scoping review allows assessment of current areas of research, as well as gaps in knowledge. This scoping review aims to systematically identify and map the current literature on buprenorphine for pain management in children with SCD to clarify what is known and highlight gaps for future research. We anticipated that our scoping review would identify a recent increase in publications concerning the use of buprenorphine for pediatric SCD patients, but that the publications would be limited in scope, restricted to case reports and smaller cohort studies. We also anticipated that, while generally supportive of buprenorphine use for SCD pain, their results would likely be difficult to generalize from the available data. We also anticipated that qualitative analysis would show that publications so far have tended to focus on themes of “efficacy” and “patient experiences” in their discussion of buprenorphine for pain management.

## 2. Materials and Methods

**Figure A1:**
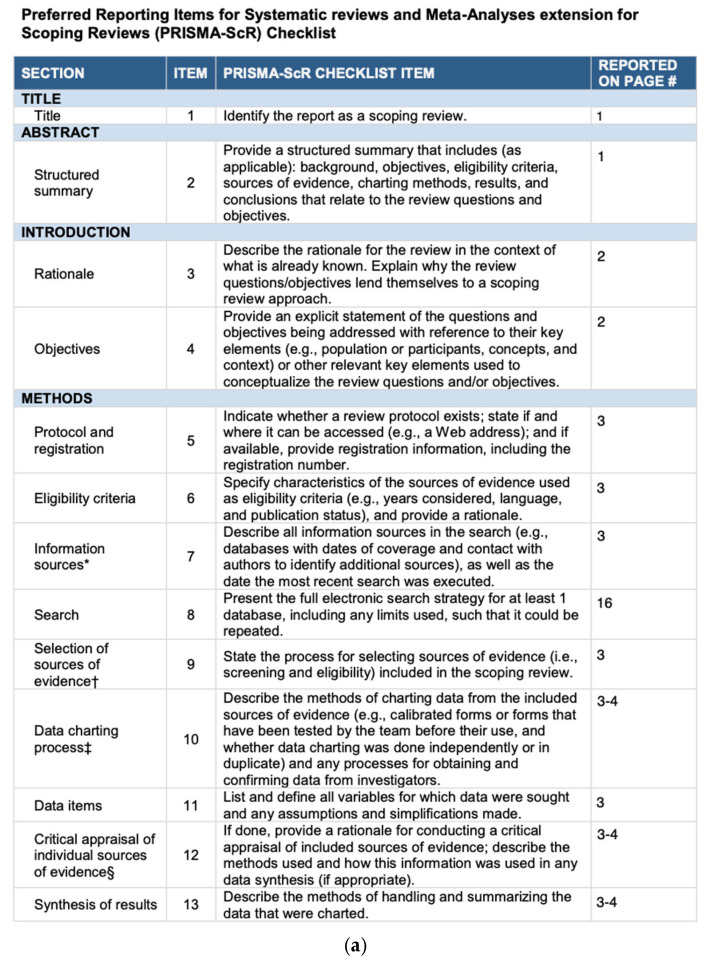

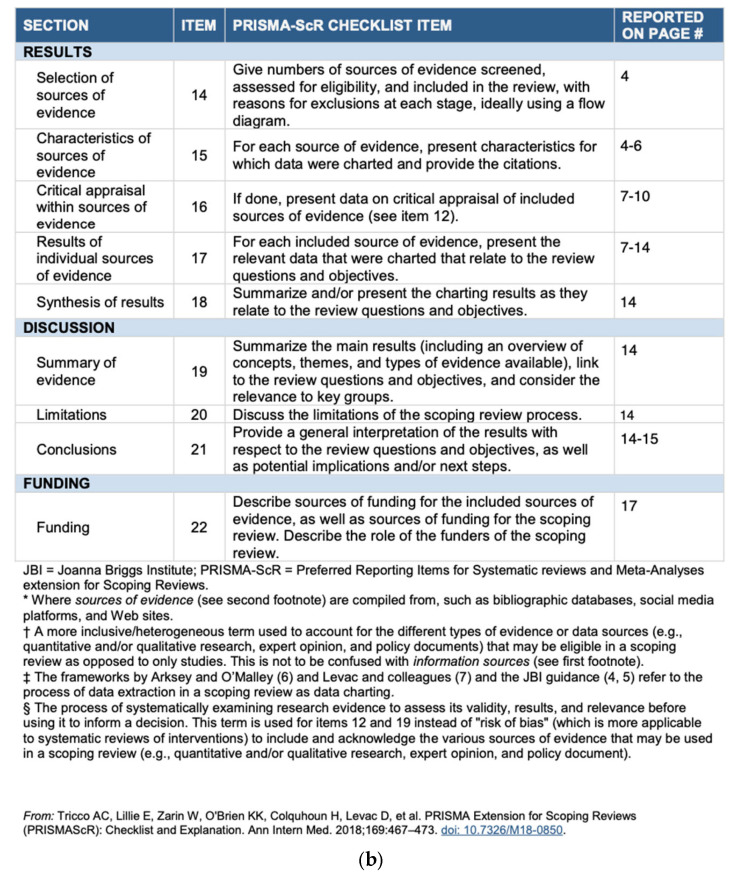
(**a**,**b**) Completed PRISMA—ScR Checklist, available from the PRISMA Extension for Scoping Reviews website [17].

This scoping review was conducted in accordance with the JBI methodology for scoping reviews and in accordance with the Preferred Reporting Items for Systematic Reviews and Meta-Analyses (PRISMA) guidelines and the PRISMA extension for Scoping reviews (PRISMA-ScR) [15,16,17]. The full PRISMA-ScR Checklist can be found in Appendix B, Figure A1. A preliminary search of PubMed, the Cochrane Database of Systematic Reviews, Open Science Framework (OSF) Registries, and Joanna Briggs Institute (JBI) Evidence Synthesis was conducted; no current or ongoing systematic reviews or scoping reviews on alternative pain management for children with SCD were identified, and no reviews specifically on buprenorphine use for children with SCD were found. An a priori protocol was then designed for this study; the study was then registered with the Open Science Framework (registration https://doi.org/10.17605/OSF.IO/WATY6 (accessed on 9 February 2026)) [18]. This scoping review aimed to identify studies/evidence for pediatric patients (younger than 21 years of age at beginning of the study) with SCD (excluding those with sickle cell trait) and use of buprenorphine for pain management (acute or chronic).

### 2.1. Search Strategy

The search was conducted in September 2025 using PubMed (MEDLINE), Embase, and Web of Science. Controlled vocabulary terms and their entry terms were used to inform the final keyword list. The search strategy included keywords related to sickle cell anemia and buprenorphine, including but not limited to “sickle cell,” “hbs disease,” “buprenorphine,” and “suboxone,” along with keywords covering a pediatric and young adult population (See Appendix A). No date range was used, and only studies in English were included.

### 2.2. Study/Source of Evidence Selection

Following the search, all identified citations were collated and uploaded into Covidence review software (Veritas Health Innovation, Australia 2025), and duplicates were removed. Titles and abstracts were screened by two independent reviewers (J.D. and M.N.) for assessment against the inclusion criteria for the review. Any disagreements between reviewers during the screening were resolved by a third reviewer (N.A.), who is a subject matter expert in pediatric hematology.

**Figure 1:**
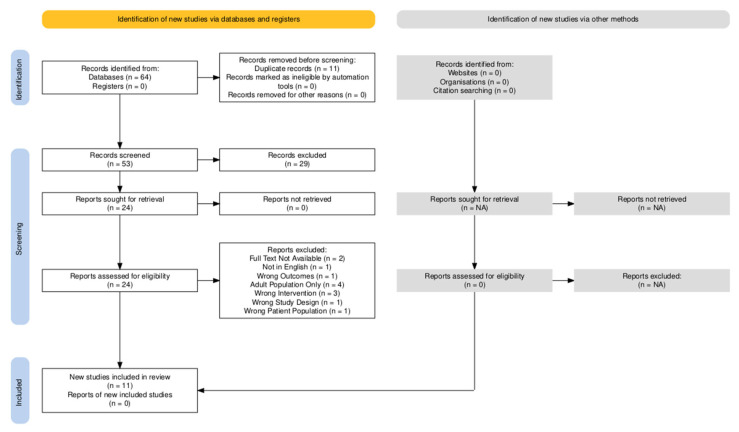
PRISMA flow diagram (diagram created with use of the Shiny app for producing PRISMA flow diagrams [21]).

Potentially relevant sources were then reviewed in full by at least two reviewers. Disagreements between the reviewers during full-text review were resolved through discussion. Reasons for exclusion of sources of evidence during full-text review and the full consort diagram are included in Figure 1.

### 2.3. Data Extraction

For this review, data was extracted by two or more independent reviewers (J.D., M.N., and N.A.) using Covidence review software to ensure rigorous and systematic data collection. Reviewers used a standardized extraction form to capture key elements, including the author(s), year of publication, country or region, study type and design, sample size (if applicable), age range, sickle cell phenotype (if available), indications and regimens for buprenorphine use, outcomes assessed, primary findings, and any stated limitations or conflicts of interest. Discrepancies between reviewers were resolved by consensus, maintaining reliability and reducing bias in the data extraction process [15].

In addition to quantitative synthesis, qualitative thematic analysis was performed informed by JBI guidance for scoping reviews. We used a two-staged approach for qualitative data extraction and synthesis. Prior to screening and data extraction, the review team developed an a priori thematic framework that consisted of a pre-specified deductive thematic analysis and a post hoc inductive thematic assessment. We agreed on a set of anticipated themes (i.e., “Efficacy”, “Comparative Effectiveness”, “Provider Experiences”) to guide initial data extraction. During full-text review, each study was assigned one major theme, and at least one minor theme. Disagreements between reviewers of the main themes were resolved through discussion. Reviewers also collected exemplary quotes from these studies, and qualitative themes were identified in a post hoc thematic assessment, with reviewers independently coding these quotes and determining themes through a consensus decision [19,20]. The thematic synthesis also identified and collated key areas identified for future research. The reviewers conducted the coding independently, with regular meetings to ensure consistency and resolution of disputes through discussion.

## 3. Results

Using a Boolean expression developed with the assistance of the Boston Children’s Hospital Library, our search identified 64 unique pieces of evidence at the time of collection (29 September 2025) from three databases. No current studies or protocols were identified in our search of registries. Of these studies, 11 duplicates were removed, and 53 studies were included in the screening process. During abstract review, 29 studies were removed, either for having a topic unrelated to our study question or for specifically stating that sickle cell disease was an exclusion criterion for the study (see PRISMA flow diagram in Figure 1). Of the studies that completed a full-text review, the most common reason for exclusion was that the study was restricted to an adult population followed by “wrong intervention”, i.e., buprenorphine use was not being studied or reported on (see Figure 1).

### 3.1. Quantitative Analysis

Of the pieces of evidence included, all except one, Admiraal, M., et al., 2023, were from the United States (US) [22]. One piece was published in 2010, with the remainder published in the last five years (from 2020 onwards). The majority were case reports (6) describing the use of buprenorphine for children or young adults with SCD, with chronic pain not well controlled by full-mu agonist opioids [23,24,25,26,27,28]. There were three retrospective analyses, which reported on patient outcomes using buprenorphine regimens in the inpatient and emergency room setting, and two cross-sectional studies, which assessed provider experience and knowledge about buprenorphine use for patients with SCD [12,22,29,30,31] (see Table 1).

### 3.2. Buprenorphine Strengths and Formulations

Buprenorphine is a potent analgesic; when given parenterally, it is roughly 30 times more potent than morphine [32]. It is heavily metabolized by the liver and, due to this, has poor oral bioavailability [33]. There are currently no approved oral formulations, but it can be administered through various routes. The most common pain management applications include intravenous (IV), transdermal, sublingual (tablets or film), and buccal film. Other available forms include intramuscular, long-acting injectable, and intranasal application [34]. The sublingual tablet/film and buccal film formulations are available with or without naloxone, an opioid antagonist. Combination dosing has little effect on medication efficacy, and has been added to decrease diversion and misuse [35]. As there are a number of different formulations, we have summarized common available formulations and the adult dosing and pharmacokinetic data in Table 2, with pediatric dosing provided for the IV formulation.

In the studies identified, sublingual was the most common route. One study, ref. [22], used the IV formulation of buprenorphine, while the other studies used sublingual formulations of buprenorphine either alone or in combination with transdermal or buccal formulations (see Table 1). The two studies that used transdermal patches both used them for induction with the goal of rotating to the sublingual or buccal formulation [12,26].

### 3.3. Case Reports

A common element of the case reports included in our review was patients with difficult-to-treat pain, often with several co-morbidities. Osunkwo et al., in a symposium abstract [23], describes an 18-year-old female with SCD (genotype Hemoglobin SS or HbSS), recurrent strokes, and moyamoya, as well as recurrent inpatient admissions for pain. She received multi-modal therapy, including cognitive behavior therapy, and a medication regimen that included gabapentin, topiramate, clonidine, and amitriptyline. Buprenorphine was used to wean her off all narcotic medications, including buprenorphine. The abstract only reported the 5 days following discontinuation of buprenorphine outpatient, but stated that at that time, pain was well controlled off opioids. Jacob and Chang, in their 2022 abstract at the American Society for Pediatric Hematology/Oncology Conference, reported a planned admission for a 16-year-old female with HbSS who, after 6 long admissions during a single year, was admitted for a multidisciplinary, regimented admission with daily psychological and physical therapies, continuous opioid infusions and medication titration. After 4.5 weeks of hospitalization, this process led to a home regimen of buprenorphine, amitriptyline, and pregabalin. They reported no admissions for 3 months following this admission, with only occasional acute care visits [25].

Irwin et al. describes an 11-year-old female with SCD (Hemoglobin SC genotype or HbSC) who, at the time of the report, had been admitted 11 times in the prior 7 months with high outpatient opioid use [24]. Her oral morphine equivalents (OMEs), a standardized estimated equivalency used to compare opioid doses, was high, with roughly 80 mg OMEs from hydromorphone in addition to a daily methadone dose. She was admitted for a rotation to buprenorphine and experienced significant complications, including admission to the intensive care unit. On discharge, she was still taking hydromorphone in addition to her buprenorphine, though the hydromorphone was weaned off slowly in the outpatient setting. The authors of this paper specifically note that while buprenorphine appeared to be effective in controlling this patient’s pain, the “relative lack of research into best practices, efficacy, and safety of buprenorphine for pediatric chronic SCD pain” was a barrier to its use, and as evidenced by their experience, the process of transition can be complicated. A case series from Osunkwo et al. in 2020 is included here; only one of the two patients in this case series met our inclusion criteria for age, as they were under the age of 21 at the beginning of their report [27]. The report describes this patient’s care from adolescence through a buprenorphine rotation at the age of 25 years. While this series is limited in its generalizability to our patient population, it provides some more details about the clinical decision-making process and discusses the importance of recognizing the role of neuropathic pain in SCD-related pain as well as mental health concerns.

Our strongest qualitative statements on buprenorphine’s effect came from Leyde et al., where they reported on a 22-year-old (included here because the case report follows her case from the age of 20 years) with SCD who experienced significant hardship including incarceration in addition to frequent difficult-to manage pain episodes [26]. The patient was on chronic opioid therapy (COT) for her pain, taking 160 mg every 6 h (960 mg OME), and during an admission for pain where she was receiving continuous IV opioids, through shared decision making, the medical team started a microdose induction with buprenorphine, where they increased from a relatively low dose up to her intended outpatient dose with a combination of transdermal patches and sublingual tablets. She was able to wean off full μ-opioid agonists to a buprenorphine/naloxone combination sublingual tablet and reported complete resolution of her pain in the outpatient setting. The report provides quotes from both the patient and providers such as, “With the oxy [oxycodone] I just built up a tolerance which the meds stop effecting me pain wise and was only giving a sleepy/mood swings side effect […] With buprenorphine I feel more energy, more clearheaded and some sort of relief”, providing a clear example of patient perceived satisfaction.

Buchheit et al. report on an ambulatory regimen for two adolescent patients with HbSC who initiated buprenorphine/naloxone for pain control [28]. This series provides the exact wean/cross-titration regimen used for each patient. Neither required an admission for this rotation and both reported subjective measures of improved pain management and quality of life, though no standardized measures were included in this report. There is some limited patient-level data on healthcare utilization that was extracted and is included in Table 3. It again points to a lack of data or existing regimens for buprenorphine induction in this patient population but provides support for the idea that, like adult data, it may have similar risks and benefits.

### 3.4. Retrospective Analyses

The most applicable and generalizable retrospective study included comes from Patel et al., which describes the use of a microdose induction regimen of buprenorphine for adolescents with SCD whose pain was not controlled on COT [12]. This analysis reported on 14 patients and provides a detailed protocol including pre-screening recommendations, supportive care medications, and dosing regimens for buprenorphine, which could be replicated in other settings. This protocol was designed as an inpatient induction, and most patients were induced during an unplanned admission for pain. This study showed that, of the patients rotated, there was a large decrease in acute care utilization and inpatient admissions and that this micro-induction protocol was safe and effective. The majority of patients remained on buprenorphine at the time of reporting and subjectively reported improved quality of life, though the authors note that they did not follow quality-of-life measures in any objective manner.

Admiraal et al. and Sabharwal et al. [22,29] conducted retrospective analyses that looked at an emergency department-initiated protocol for patients presenting with SCD-related pain and a protocol for rotating palliative care patients, including those with SCD, to buprenorphine, respectively. While both included pediatric patients with SCD and the use of buprenorphine, their respective goals were different than other studies reported here. Admiraal et al. was focused on the development of protocol to initiate pain control for SCD patients on their arrival to an emergency department. Buprenorphine, in the intravenous formulation, was included as an alternative for morphine for patients with previous adverse reactions to morphine. Buprenorphine was not compared against morphine, but they do not report significant differences between the two medications and their efficacy. Sabharwal et al. aimed to show that buprenorphine can be used effectively for pain management and often at lower doses than full μ-opioids; they stated that their general protocol is to consider buprenorphine for patients requiring more than 100 mg OME daily and to rotate them to low-dose buprenorphine, rarely exceeding 6 mg per day. The authors suggested that for many patients, this resulted in an equianalgesic effect. There is no sub-group analysis of the patients with SCD, making it hard to generalize the results of this study to our patient population.

### 3.5. Cross-Sectional Analyses

Two cross-sectional analysis are included, which are different analyses of the same survey, one by Tiako et al. and the other by Afranie-Sakyi et al. [30,31]. The survey was provided to physicians and advanced practice providers caring for children or adults with SCD in the United States (US), who were recruited using SCD-focused listservs. The data provided is limited: we are not given a response or completion rate, the two studies show responses for the same group of participants, and due to the self-selecting nature of this survey, the limited population of providers included may not be indicative of providers more generally. The two reports have slightly different focuses but indicate that providers, while aware of buprenorphine as a potential medication for SCD-related pain, may need more education on its prescribing and safety/side effects, particularly in pediatric providers.

### 3.6. Patient Level Data

A subset of these reports provided us with patient-level data pre and post initiation of buprenorphine. Assessing the data collected/provided in these case reports and retrospective analyses, they included several different metrics to define successful use of buprenorphine for pain management. These metrics included: change in oral morphine equivalents (OMEs), change in acute care utilization, change in number of hospital admissions, change in hospital length of stay (LOS), and often a statement of qualitative improvement, though none used a specific standardized metric to assess improvement in quality of life. For studies where patient-level data was available for our population of interest, we created a collated table of data to show their reported outcomes (see Table 3).

From this quantitative data, we can see that all of the patients on average have high daily OMEs, and patients in the case reports tended to have higher reported OMEs than those included in the retrospective analysis (the average for the retrospective analysis was 24 mg OMEs versus OMEs greater than 100 mg in most cases listed above). All report a decrease in their OMEs, acute care utilization, hospital admissions, and total number of hospital days. Buprenorphine doses varied, but patients tended to be on sublingual formulations (either film or tablet) with a dose as low as 1 mg daily and as high as 10 mg QID. It is difficult to generalize data across these various studies, but they suggest that rotation to buprenorphine may result in a decrease in acute care and hospitalization for selected patients, and for pediatric patients, buprenorphine may be effective in managing SCD-related pain.

### 3.7. Qualitative Assessment

**Figure 2:**
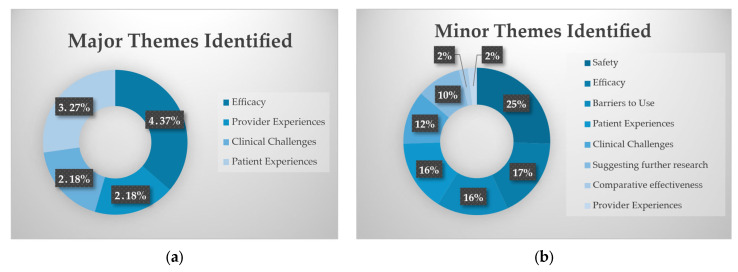
Themes identified in the studies based on our pre-determined list of themes: (**a**) major themes identified, total number of times a theme was identified and percentage frequency of that theme; and (**b**) minor themes identified and percentage frequency of those themes.

Each study included had a major qualitative theme, and up to 3 minor qualitative themes identified. Each reviewer independently identified the major and minor themes based on a predetermined set of possible themes. Conflicts about the major themes of the studies were resolved by discussion among the reviewers, and consensus was reached through an iterative process. The most common major theme identified for these was “Efficacy” followed by “Patient Experiences”. For minor themes, “Safety” was the most identified theme, followed by “Efficacy” (see Figure 2). These themes are reflective of the fact that most of the included papers were case reports, showcasing episodes of successful use of buprenorphine. Notably “Comparative Effectiveness” was only identified once as a minor theme by the reviewers, which demonstrates that while these papers were overall positive in their report of buprenorphine, very few specifically commented on how effective it was in comparison to other treatments or discussed other options for treatment besides buprenorphine.

In addition to our predetermined theme identification, authors were asked to identify key quotes from the studies. The review team then met to review these quotes and identify consensus themes that may not have been captured by our initial thematic analysis. Of the key quotes pulled from the studies, a number (8 of 32 quotes) fell into our predetermined category of “Patient Experiences”; however, the post hoc analysis also identified “Need for Guidance/Guidelines” as an important theme in these studies (see Table 4).

Our review team also collected areas identified for future study. The need for prospective trials, education, and development of guidelines to manage sickle cell pain in pediatric patients were the most commonly identified areas in need of future study. A full table is available in Appendix A (Table A3).

## 4. Discussion

Our scoping review of the literature on buprenorphine use in pediatric patients with sickle cell disease shows that data in this area is overall of low quality and limited in scope. There are few published studies on this topic and no randomized controlled trials of any kind. Our review identified a small sample of studies and case reports that suggest that rotation to buprenorphine from full μ-opioid agonists may be effective for managing SCD-related pain and may improve clinical outcomes for children with SCD and chronic pain. There is a significant need for more guidance on the appropriate use of this medication. A survey of providers reported that a majority would like to receive education on how to initiate patients on buprenorphine, and discomfort with dosing was the most common barrier to prescription for providers, suggesting that additional education is also needed [30]. One of the retrospective studies [12] provided a detailed framework and protocol for the rotation of patients from full-agonist opioids in the inpatient setting, and one case series provided the titration plan utilized [28], but further study is required to validate these protocols in other settings and institutions.

There is limited patient-level data, but rotation to buprenorphine for chronic SCD pain has been suggested to decrease acute care utilization, decrease hospitalization rates, and decrease OMEs while improving reported pain. There are significant limitations to this review, including a lack of randomized controlled trials and heavy reliance on case reports, which have an inherent reporting bias; times when patients were rotated to buprenorphine unsuccessfully were not represented in these studies. Additionally, the patient population represented in these studies is skewed towards patients who may be interested in starting buprenorphine and feel that it would likely be beneficial to start with. The majority of patients included in our analysis were female, which is consistent with previous studies showing that female patients with SCD account for the majority of inpatient admissions in the US, but this sample is not large enough to make conclusions about gender differences in this context [40].

As hypothesized, our scoping review identifies that while there has been a recent increase in published reports and abstracts about buprenorphine use for pediatric patients in the last decade, suggesting increased interest, the pediatric cases reported here note decreased acute care utilization and qualitative statements of support from providers and patients, similar to that seen in larger adult studies. The limited evidence provided here, in combination with concordance with adult data, provides strong support for future investigation of buprenorphine for SCD pain.

More detailed pharmacokinetic research in pediatric patients is also needed. In Table 2, we noted that IV/IM buprenorphine is approved in children. It has been shown to be efficacious, but the clearance is higher in pediatric patients, likely due to their higher ratio of hepatic weight to body weight [41,42]. Pharmacokinetics of transdermal, sublingual, and buccal formulations may be different in children as well, and understanding these properties could facilitate approval of other formulations for younger age groups.

The value of SCD-specific guidelines and protocols for pain management in children cannot be understated. Looking to adult studies in this area, the publication of protocols for rotation of adult patients to buprenorphine allowed the creation of follow-up studies and additional use and implementation of this medication [5,10,43]. Patel et al. provided the clearest protocol noted in this review, which could be adapted for use at other institutions; while limited to the inpatient setting, this could provide a basis for future studies to validate its use and better understand the effect on quality-of-life measures while identifying patients who would most benefit from the intervention [12]. Development of guidelines for transitioning to buprenorphine and regimens for outpatient induction would likely provide education and support for clinicians interested in considering this medication.

Protocols are particularly important for buprenorphine, with its multiple formulations and routes of administration, which may complicate initiation. Buprenorphine may also induce withdrawal in patients on COT, which increases the risk faced by patients when transitioning, and pediatric-specific protocols could help mitigate this risk. While it has been approved and shown to be effective in its IV/IM formulation, a better understanding of its pharmacokinetics in pediatric patients is needed to understand its efficacy and side-effect profile. Reducing the risk of respiratory depression by using buprenorphine may be more significant in pediatric patients as they are more likely to develop and to die from acute chest syndrome, a serious pulmonary complication that can be triggered by hypoventilation [14]. The development of guidelines, increased education about buprenorphine, and validation of protocols for its use are needed to improve the utilization and understanding of this potentially beneficial medication.

## 5. Conclusions

SCD-related pain is a significant driver of patient distress, healthcare cost, and ultimately a reflection of poor disease control [2]. While some patients will do well with disease-modifying treatments such as hydroxyurea, there are those who will continue to have significant pain despite continuous use and some for whom hydroxyurea is not indicated (i.e., patients with the HbSC genotype). For patients with frequent acute pain episodes and chronic pain related to sickle cell disease, there are limited options and limited consensus recommendations. Many patients rely on COT for chronic pain, which is associated with significant side effects and, for many patients, is often ineffective even at high doses. Patients also experience acute pain exacerbations, which similarly can be difficult to manage with traditional full μ-opioid agonists. In our limited sample, the patients noted in these case reports had high OMEs, putting them at significant risk of opioid-related side effects, which suggests that there likely is a population for whom COT alone will not control acute or chronic SCD-related pain and for whom alternative therapies are needed. Buprenorphine has potential to provide pain relief for these patients and to decrease the risk of side effects such as respiratory depression and overdose, but to increase its utilization in pediatrics will require additional studies to understand how best to implement its use for this unique population. This view is shared by the American Society of Hematology (ASH), who included “comparative-effectiveness studies between full agonist opioids and partial agonist opioid therapy, such as buprenorphine” as a research need in their 2020 guidelines for sickle cell pain management [44]. This scoping review highlights the need for a better understanding of the use, efficacy, and optimal dosing of buprenorphine for SCD-related pain. The SCD community would benefit greatly from either real-world data in the form of retrospective analyses or the development of pediatric-specific trials for sickle cell pain therapeutics, particularly aimed at patients with difficult-to-treat and refractory SCD-related pain.

## Figures and Tables

**Table 1:** Details of studies included in final scoping review.

First Author	Number of Participants	Setting	Buprenorphine Formulation	Summary
Osunkwo, I et al. 2010 [23]	1	Inpatient	Not specified	Pain not responsive to chronic opioid therapy should prompt consideration of alternate therapies, including buprenorphine.
Irwin, M et al. 2021 [24]	1	Inpatient	Sublingual	Buprenorphine proved to be a safe and efficacious alternate to chronic opioid therapy with full-agonists.
Jacob, P et al. 2022 [25]	1	Inpatient	Sublingual	Inpatient multimodal intensive pain management with buprenorphine is successful at decreasing acute care utilization and hospitalizations.
Leyde, S et al. 2022 [26]	1	Inpatient	Transdermal and sublingual	Inpatient microdose induction during a pain episode is feasible and safe and may lead to improved outcomes over full-agonist opioids.
Osunkwo, I et al. 2020 [27]	1 *	Outpatient	Sublingual buprenorphine/naloxone	In combination with increased psychosocial support, buprenorphine can be an effective for transitioning patients off chronic opioid therapy.
Buchheit, B 2021 [28]	2	Outpatient	Sublingual buprenorphine/naloxone	Ambulatory buprenorphine/naloxone induction is feasible and tolerable in adolescent patients and may lead to improved quality of life after transition.
Admiraal, M et al. 2023 [22]	131	Emergency Room	Intravenous	Implementation of a standardized pain protocol resulted in overall shorter time to first dose of opioid. Length of stay and admission rates were comparable to published averages.
Sabharwal, B et al. 2024 [29]	19	Inpatient	Sublingual	For patients on high doses of opioids, low-dose buprenorphine can be equally efficacious.
Patel, A et al. 2025 [12]	14	Inpatient	Transdermal patch, sublingual or buccal	Micro-dose induction followed by buprenorphine maintenance dosing is both safe and effective for use in pediatric patients with SCD and chronic pain.
Afranie-Sakyi, J et al. 2024 [30]	52 †	Not applicable	Not applicable	Many providers do not feel comfortable prescribing buprenorphine, particularly pediatric providers.
Tiako, M et al. 2024 [31]	52 †	Not applicable	Not applicable	Providers have some baseline knowledge about buprenorphine but may be unsure about dosing and prescribing.

* Two patients were reported in this study but only one met the inclusion criterion: being younger than 21 years of age at the time of enrollment/beginning of the report. † These two studies report data from the same set of survey responses.

**Table 2:** Adult data on selected buprenorphine formulations, including available formulations, dosing, and key pharmacokinetic parameters relevant to pain management.

Formulation	Brand(s) (US) *	Common Dosages Available *	Typical Starting Dose for Pain (Adult Unless Specified) †	Time to Peak Plasma Concentration ^‡^	Mean Half-Life ^‡^
Transdermal patch (7-day)	Butrans, generic	5, 7.5, 10, 15, and 20 mcg/h patches	FDA-approved for chronic pain; opioid-naïve or <30 mg oral MME/day: 5 mcg/h once weekly; 30–80 mg MME/day: 10 mcg/h once weekly, not to exceed 20 mcg/h.	72 h to steady state	~26 h after patch removal
Buccal film	Belbuca	75, 150, 300, 450, 600, 750, and 900 mcg films (q12 h dosing)	FDA-approved for chronic pain; 75 mcg once daily or q12 h for ≥4 days, then increase, not to exceed 900 mcg/12 h.	2.5–3 h	~27–28 h
Buccal/sublingual film (buprenorphine/naloxone)	Generic	2 mg/0.5 mg, 4 mg/1 mg, 8 mg/2 mg, and 12 mg/3 mg	Off-label for pain, with a typical starting dose of 2 mg/0.5 mg §	Not available	16.4–27.5 mg (buprenorphine); 1.9–2.4 mg (naloxone)
Injection IV/IM	Buprenex, generic	0.3 mg/mL solution in 1 mL	FDA-approved for acute pain in children >2 years of age; acute pain (>2 y <12 y) 2–6 mcg/kg q4–6 h; acute pain (≥13 y): 0.3 mg IV or IM q6 h PRN; or 2–6 mcg/kg for children q4–6 h.	IV: rapid; IM: ~60 min	1.2–7.2 h (may be shorter in children)
Sublingual tablet	Zubsolv, Subutex, generic	2 and 8 mg	Off-label for pain, with a typical starting dose of 2–4 mg q6–8 h §	1.3–1.8 h	31–35 h
Sublingual tablet (buprenorphine with naloxone)	Suboxone, generic	2 mg/0.5 mg and 8 mg/2 mg	Off-label for pain, with a typical starting dose of 2–4 mg q6–8 h §	24–42 (buprenorphine); 2–12 (naloxone)	Not specified

**Pediatric dosing is off-label except for IV/IM formulations at the time of publication, and data cannot be extrapolated to pediatrics; the chart is included for reference and background purposes.** Transdermal and buccal formulations are dosed in micrograms; sublingual formulations are dosed in milligrams and are not dose-equivalent. Starting doses reflect adult label-based guidance.* Drug dosage and formulation information was adapted from Jonan et al. 2018 and Adler et al. 2024, as well as data available from UpToDate^®^ Lexidrug™ [36,37,38,39]. † Starting doses are available either from monographs at UpToDate^®^ Lexidrug™, Johnson et al. 2005, and Adler et al. 2024, or, for sublingual combination tablets, David et al. 2022 [5,32,37,38,39]. ‡ Pharmacokinetic data is available in Jonan et al. 2018 and in drug monographs through UpToDate^®^ Lexidrug™ [36,38,39]. § Based on Buccheit et al. 2021, if a patient is cross-titrating off full-mu opioid doses as low as 0.5 mg, buprenorphine may be used [28].

**Table 3:** Patient-level outcome data reported in selected studies: (a) shows baseline characteristics provided in these studies; and (b) shows changes reported pre and post induction to buprenorphine.

(a)					
Study (Year)	Genotype	*N*	Age (Years)	Sex	Baseline Opioid Dose
Osunkwo et al., 2010 [23]	HbSS	1	18	F	Methadone 270 mg/day
Osunkwo et al., 2020 [27]	HbSS	1	25	F	120 mg OME/day
Buchheit et al., 2021 (Case 1) [28]	HbSC	1	17	M	Methadone 12.5 mg/day + oxycodone 37.5 mg/day
Buchheit et al., 2021 (Case 2) [28]	HbSC	1	19	M	270 mg OME/day (oxycodone)
Irwin et al., 2021 [24]	HbSC	1	11	F	Methadone 5 mg TID + hydromorphone 4 mg PO every 4 h
Patel et al., 2025 [12]	12 HbSS; 2 HbSβ^0^	14	18 (14.8–18.9)	9 F; 5 M	24.4 mg OME/day (IQR 18–40)
(**b**)					
**Study (Year)**	***N***	**Pre-Intervention Healthcare Utilization**	**Post-Intervention Opioid/Buprenorphine Regimen**	**Post-Intervention Healthcare Utilization**	**Follow-Up/Notes**
Osunkwo et al., 2010 [23]	1	Single 90-day hospitalization	Buprenorphine during admission; 0 opioid dose at discharge	No acute exacerbations requiring admission	6 months; methadone stopped by day 65, buprenorphine stopped by day 85
Osunkwo et al., 2020 [27]	1	25 ED visits/year; 5 admissions/year; mean LOS 3 days	Sublingual buprenorphine/naloxone (dose not specified)	No ED visits	12 months; authors report acute care use “dropped dramatically”
Buchheit et al., 2021 (Case 1) [28]	1	High ED utilization; estimated 13–17 ED visits/year	Buprenorphine/naloxone SL 2 mg/0.5 mg TID → 1 mg/0.25 mg SL *	No ED or acute care visits	5 months; improved pain reported
Buchheit et al., 2021 (Case 2) [28]	1	6 admissions/year; 63 inpatient days/year	Buprenorphine/naloxone SL 4 mg/1 mg TID → 4 mg/1 mg film daily *	Fewer acute care visits (number not reported); 1 admission (11 days)	6 months
Irwin et al., 2021 [24]	1	1.57 hospitalizations/month (11 admissions/7 months)	Buprenorphine/naloxone SL films titrated to 2 mg QID (8 mg/day) + hydromorphone PRN (OME 96 mg/day *)	0.86 hospitalizations/month (6 admissions/7 months)	OME excludes buprenorphine; full-agonist opioids weaned to PRN
Patel et al., 2025 [12]	14	Median 11 acute care encounters/person-year	Transdermal buprenorphine 10 → 20 mcg/h, then SL/buccal buprenorphine or buprenorphine/naloxone; median discharge dose 8 mg/day (range 2.5–16)	Median 8 acute care encounters/person-year; median decrease of 3.5 inpatient admissions/person-year; median decrease of 40 hospital days	12 months; 3 patients discontinued buprenorphine

(**a**) Age and baseline opioid dose are reported as presented in the original studies (mean, median, range, or regimen). OME values reflect full-agonist opioids only when specified by authors. Buprenorphine doses are not included in baseline opioid calculations. (**b**) Outcomes are reported as presented in the original studies. Due to heterogeneity in outcome definitions, follow-up duration, and reporting metrics, data were not normalized, pooled, or imputed. OME values exclude buprenorphine and reflect full-agonist opioid use only, as specified by study authors. Rates, medians, and ranges are retained when raw counts were not provided. * In these cases, the patients had and initial maintenance dose that was adjusted based on symptoms and the dose after the arrow indicates their final maintenance dose OME values reported by authors; post-intervention OME reflects PRN opioid availability rather than scheduled dosing.

**Table 4:** Exemplar quotes and example of thematic analysis of that quote.

Number of Quotes	Themes Identified
*N* = 32	
8	Patient Experiences
6	Barriers to Use
4	Need for Guidance/Guidelines
2	Increased Interest
2	Provider Experiences
2	Equivalency of Dosing/Effect
2	Knowledge Gap
2	Need for Multimodal Therapy
1	Clinical Challenges
1	Need for Multidisciplinary Team
1	Efficacy
1	Feasibility
Exemplar Quotes: Patient Experiences	
Citation	Quote
Leyde, S et al. 2022 [26]	[Patient reflection] “With the buprenorphine I feel more energy, more clear-headed and some sort of relief … I feel that the switch was very helpful and necessary. I would recommend it for anybody.”
Buchheit, B 2021 [28]	“…[The patient, after rotation to buprenorphine] consistently reports feeling like a normal teenage, and plans to return to playing soccer.”
Exemplar Quotes: Need for Guidance/Guidelines	
Citation	Quote
Jacob, P et al. 2022 [25]	“Despite a high prevalence of CPS [Chronic Pain Syndrome] in pediatric patients with SCD, there is a paucity of standardized management tools and a scarcity of resources to address chronic pain in the SCD population.”
Sabharwal, B et al. 2024 [29]	“What is missing is a more clear understanding of the potential conversion ratios to better target dosing for patients with pain rotated to buprenorphine using a low dose initiation strategy”

## Data Availability

Documents containing data collected by individual reviews and the collated data is available on request.

## Abbreviations

The following abbreviations are used in this manuscript:

SCD	Sickle Cell Disease
OSF	Open Science Framework
JBI	Joanna Briggs Institute
OME	Oral Morphine Equivalent
PCA	Patient-Controlled Analgesia
BID	Twice daily (bis in die)
HbSS	Homozygous Sickle Cell Disease Genotype “SS”
HbSC	Compound Heterozygous Sickle Cell Disease Genotype “SC”
HbSβ-	Sickle-Beta naught Thalassemia
US	United States
COT	Chronic Opioid Therapy
LOS	Length of Stay
ASH	American Society of Hematology

## Appendix A

### Appendix A.1. Boolean Search Strategy

Our study focuses on pediatric patients with sickle cell treated with buprenorphine. Using MeSH terms and their related keywords, we were able to create a list of keywords for our Boolean search phrase.

**Table A1:** Keywords/synonyms used in our Boolean phrase.

Sickle Cell	Buprenorphine	Pediatrics
“sickle cell”	buprenorphine	pediatric*
“Hemoglobin S Disease*”	acimaphin	newborn*
“Sickling Disorder”	suboxone	neonat*
“HbS Disease”	subutex	infant*
drepanocytemia		child*
“drepanocytic anaemia”		adolescent*
“drepanocytic anemia”		teen*
drepanocytosis		“young adult*”
“haemoglobin SS”		pediatric*
“haemoglobin SS”		

This resulted in the following Boolean search phrase, which was used to identify studies for our scoping review:

(“sickle cell” OR “Hemoglobin S Disease*” OR “Sickling Disorder” OR “HbS Disease” OR “haemoglobin SS” OR “haemoglobin SS”) AND (buprenorphine OR acimaphin OR suboxone OR subutex) AND (pediatric* OR newborn* OR neonat* OR infant* OR child* OR adolescent* OR teen* OR “young adult*” OR paediatric*).

### Appendix A.2. Qualitative Thematic Analysis

**Table A2:** Pre-specified themes identified for the analysis.

**Themes**
Safety
Efficacy
Comparative effectiveness
Barriers to Use
Provider Experiences
Patient Experiences
Clinical Challenges
Suggesting further research

### Appendix A.3. Areas Identified for Future Study

**Table A3:** Areas identified for further research.

Study Citation	Areas Identified for Further Research
Patel, A et al. 2025 [12]	Need for prospective clinical trial
Afranie-Sakyi, J et al. 2024 [30]	Need for buprenorphine education
Tiako, M et al. 2024 [31]	Need for buprenorphine education
Sabharwal, B et al. 2024 [29]	Need for guidelines on rotating patients to buprenorphine and determining equianalgesic dosing
Admiraal, M et al. 2023 [22]	Need for prospective clinical trial
Jacob, P et al. 2022 [25]	Need for standardized management strategies for patients with chronic pain
Leyde, S et al. 2022 [25]	Need for guidelines on rotating patients to buprenorphine and determining equianalgesic dosing
Irwin, M et al. 2021 [24]	Need for prospective clinical trial
Buchheit, B 2021 [27]	Need for prospective clinical trials and long-term outcome data
Osunkwo, I et al. 2020 [27]	Need for larger prospective trials
Osunkwo, I et al. 2010 [23]	Need for standardized management strategies for hyperalgesia syndrome

## Appendix B

Appendix B Preferred Reporting Items for Systematic Reviews and Meta-Analyses Extension for Scoping Reviews (PRISMA-ScR) Checklist.
